# Pretreatment with remote ischemic conditioning attenuates testicular damage after testicular ischemia and reperfusion injury in rats

**DOI:** 10.1371/journal.pone.0287987

**Published:** 2023-10-26

**Authors:** Jiaxue Li, Zhibing Yan, Qifeng Wang, Shichao Wei, Quanhua Liu, Ting Liu, Zhaoyang Hu

**Affiliations:** 1 Department of Anesthesiology, West China Hospital, Sichuan University, Chengdu, Sichuan, China; 2 Laboratory of Anesthesia and Critical Care Medicine, National-Local Joint Engineering Research Centre of Translational Medicine of Anesthesiology, West China Hospital, Sichuan University, Chengdu, Sichuan, China; Khalifa University of Science and Technology, UNITED ARAB EMIRATES

## Abstract

Testicular torsion is a urological emergency. However, surgical detorsion of the torsed spermatic cord can cause testicular reperfusion injury. Although remote ischemic preconditioning (RIPC) has been convincingly shown to protect organs against ischemia/reperfusion (I/R) injury, little is known regarding the effect of RIPC on testicular torsion/detorsion-induced reperfusion injury. Therefore, we aimed to evaluate the effect of RIPC on testes after testicular I/R injury in a rat model *in vivo*. Male Sprague–Dawley rats were randomly classified into 4 groups: sham-operated (sham), testicular I/R (TI/R), or remote liver (RIPC liver) and limb (RIPC limb) ischemic preconditioning groups. Testis I/R was induced by 3 h of right spermatic cord torsion (720° clockwise), and reperfusion was allowed for 3 hours. In the RIPC group, four cycles of 5 min of ischemia and 5 min of reperfusion were completed 30 min prior to testicular torsion. The ERK1/2 inhibitor U0126 was administered intravenously at the beginning of reperfusion (1 mg/kg). The testes were taken for the oxidative stress evaluations, histology, apoptosis, immunohistochemical and western blotting analysis. Remote liver and limb ischemic preconditioning attenuated ipsilateral and contralateral testicular damage after testicular I/R injury. For example. RIPC reduced testicular swelling and oxidative stress, lessened structural damage, and inhibited the testicular inflammatory response and apoptosis. Furthermore, RIPC treatment enhanced testicular ERK1/2 phosphorylation postI/R. Inhibition of ERK1/2 activity using U0126 eliminated the protection offered by RIPC. Our data demonstrate for the first time that RIPC protects testes against testicular I/R injury via activation of the ERK1/2 signaling pathway.

## Introduction

Testicular torsion is mostly seen in young people. When the spermatic cord twists within the scrotum, the blood flow of the testis is blocked, resulting in ischemia or permanent damage to the testicle. Emergency surgery is the only treatment option. However, the re-establishment of oxygenation and blood flow to the ischemic testis may cause further testicular ischemia/reperfusion (I/R) injury. The testicular tissue is very sensitive to oxidative stress [1]. Reperfusion may lead to the production of high levels of oxidative stress, which may impair spermatogenesis and increase male infertility or subfertility. Therefore, the identification of effective therapeutic interventions aimed at reducing testicular detorsion-induced reperfusion injury, promoting spermatogenesis, or improving testicular function is of great importance.

Ischemic preconditioning (IPC) was first proposed in 1986 by Murry et al. [2]. The intermittent ischemia/reperfusion insult produces cardioprotection against prolonged myocardial I/R injury. IPC ameliorates testicular damage after testicular I/R injury [3]. Another breakthrough discovery was remote ischemic preconditioning (RIPC), a more feasible and safe endogenous protective strategy against I/R injury [4]. RIPC can be induced in a distant organ, vascular bed, or limb, away from the site of severe ischemia. Repeated evidence has confirmed its powerful protective effects on vital organs, including the heart, lung, kidney, and liver [5]. However, it remains unknown whether RIPC can protect the testis against testicular I/R injury. Furthermore, I/R injury on the ipsilateral side can also affect the contralateral side [6]. Therefore, it is worth studying the effect of RIPC on the ipsilateral and contralateral testes. Notably, the protective effect of remote limb preconditioning has been confirmed in both preclinical and clinical studies [7]. We specifically used limb preconditioning in our study, primarily due to its clinical relevance. In addition, it has been shown that brief ischemic preconditioning stimuli executed on the liver, the largest visceral organ, also produce strong cardiac and pulmonary protection [8]. Based on these results, we hypothesize that liver preconditioning may also confer testicular protection against testicular I/R injury. Therefore, in the current study, we aimed to elucidate and compare the overall effect of remote limb or liver ischemic preconditioning on the ipsilateral and contralateral testes after testicular I/R injury.

The mechanisms underlying RIPC-induced testicular protection are completely unknown. Extracellular signal-regulated kinases (ERK1/2) is a vital member of the mitogen-activated protein kinase (MAPK) family. It is a key component in intracellular signal transduction. A variety of stimuli can activate ERK1/2 (via phosphorylation), and the phosphorylated ERK1/2 triggers many biological reactions and ultimately modulates physiological processes. A large number of studies suggest that ERK1/2 can be activated upon ischemic conditioning [9] or other protective agents such as volatile anesthetics [10]. The activation of the ERK1/2 can inhibit myocardial apoptosis and produces cardioprotection against myocardial I/R injury. In contrast, pharmacological inhibition of ERK1/2 activation could abolish the organ-protective effects offered by RIPC [8]. However, the role of ERK1/2 in RIPC-induced testicular protection against testicular I/R injury remains poorly understood.

Therefore, we aimed to determine (1) whether remote ischemic preconditioning protects the ipsilateral and contralateral testes after testicular I/R injury and (2) whether this RIPC-induced protection is mediated by the ERK1/2 signaling pathway.

## Materials and methods

### Ethical approval

The Institutional Animal Care and Use Committee of Sichuan University (Chengdu, Sichuan, China) approved all experimental protocols (Approval No: 20211199A). All rats received care in compliance with the Guide for the Care and Use of Laboratory Animals (8th edition, 2011).

### Animals

Six- to eight-week-old male Sprague–Dawley rats (weighing 200 to 220 g) were purchased from Chengdu Dashuo Experimental Animal Research Center (Chengdu, China). Rats were maintained in a standard SPF-grade facility with free access to water and food prior to experiments.

### Experimental groups

**Fig 1:**
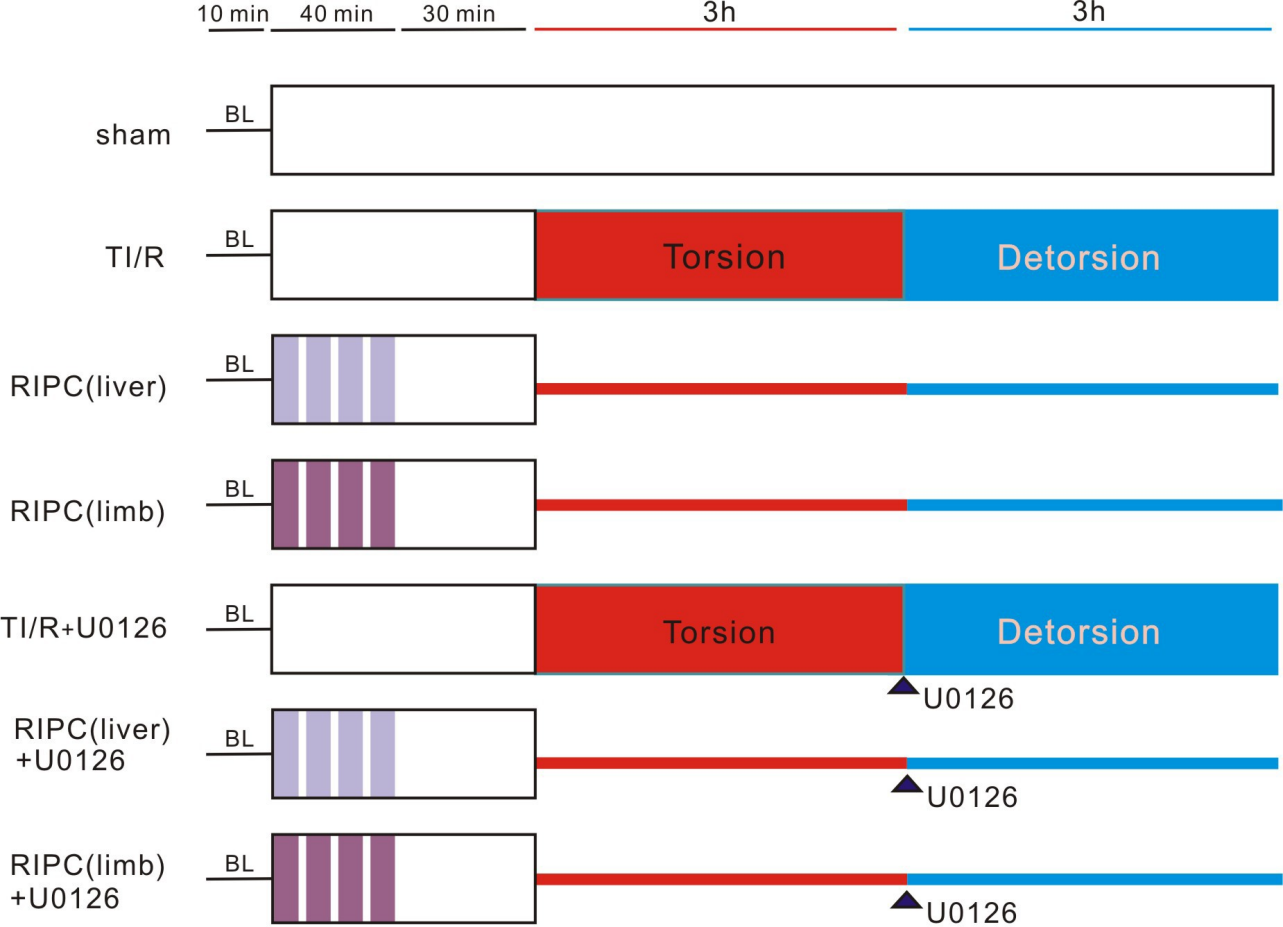
Experimental protocol. Rats in every group (except for the sham) were subjected to 3 hours of testicular torsion, followed by 3 hours of detorsion. Four cycles of intermittent ischemia/reperfusion stimuli were performed in the liver and limb ahead of testicular torsion for the induction of liver or limb ischemic preconditioning. BL: baseline; sham: sham-operated; TI/R: testicular ischemia/reperfusion; RIPC (liver): remote liver ischemic preconditioning; RIPC (limb): remote limb ischemic preconditioning. Arrow indicates the time point when U0126 was administrated.

The experimental protocol is shown in **Fig 1**. Animals were randomly assigned to the following groups: (1) Sham-operated (sham) group (animals received all procedures except for testicular torsion); (2) Testicular torsion (TT) group (rats had 3 hours of testicular torsion followed by 3 hours of detorsion); (3) Remote liver (RIPC liver) or (4) remote limb (RIPC limb) ischemic preconditioning groups: four cycles of 5-min liver or limb ischemia/reperfusion were completed 30 minutes prior to testicular torsion. Meanwhile, to elaborate the role of ERK1/2, U0126, a specific ERK1/2 inhibitor, dissolved in dimethyl sulfoxide, was intravenously injected into the rats in the TT (TT+U0126) and RIPC (RIPC liver+U0126 and RIPC limb+U0126) groups via the femoral vein 5 minutes before testicular detorsion.

### Surgical procedure and remote preconditioning

Testicular torsion and detorsion were performed under anesthesia with sodium pentobarbital (50 mg/kg, i.p.) [11, 12]. Testicular torsion/detorsion surgerical procedure was performed based on previously publications [6, 12]. Briefly, the mid-ventral laparotomy incision was performed on the ipsilateral scrotum. The ipsilateral testis was exposed and rotated 720° clockwise. After 3 hours of testicular ischemia, the testis was returned to its original anatomic position. Sodium pentobarbital (20 mg/kg, i.p.) was administered to all rats every 30 minutes to maintain anesthesia. Nalbuphine (2 mg/kg, S.C.) was used for analgesia. A thermal blanket was used to maintain the animal temperature. Three hours after testicular detorsion, rats were euthanized with pentobarbital sodium (200 mg/kg, i.p.). The ipsilateral and contralateral testes were taken, weighed and divided into two parts for determination of cellular antioxidant enzyme activities, western blot analysis, or histopathological examination. For remote liver ischemic conditioning, after laparotomy, the hepatic arterial, venous trunk and portal vein were identified. Four cycles of 5 min of hepatic ischemia followed by 5 min of reperfusion were performed using a microsurgical clamp [8]. Remote limb conditioning was conducted on the bilateral femoral arteries by four brief episodes of bilateral femoral artery occlusion (5 minutes) followed by reperfusion (5 minutes). The ERK1/2 inhibitor U0126 was administered intravenously via the femoral vein at the beginning of testicular reperfusion (1 mg/kg).

### Histological evaluation

Ipsilateral and contralateral testes were fixated in 10% neutral buffered formalin and embedded in paraffin wax. The testes were then cut into 3-μm-thick sections and stained with hematoxylin and eosin (H&E). Histological evaluation was performed under a light microscope by two independent researchers blinded to the study. The Cosentino scoring system was used to determine the degree of pathological lesions in testicular tissues [13]. The Johnson score system was used to evaluate spermatogenesis. Briefly, a score of 1 presented the worst spermatogenesis, and a score of 10 represented normal histology [14].

### TUNEL staining

Testicular apoptosis was assessed by a TUNEL staining kit (DeadEnd™ Fluorometric TUNEL System, Promega Corporation, Madison, WI, USA) according to the manufacturer’s instructions. Images were taken by a Nikon fluorescence microscope (Eclipse Ni-E, Nikon, Tokyo, Japan). The number of TUNEL-positive cells was counted from ten nonoverlapping fields from each slice. The apoptotic index was calculated as the ratio of TUNEL-positive nuclei to total nuclei.

### Testicular tissue MDA, SOD, and GSH measurement

Ipsilateral and contralateral testes were homogenized. Determination of the activities of superoxide dismutase (SOD), malondialdehyde (MDA) and glutathione (GSH) was conducted according to the manufacturer’s instructions (Jiancheng Bioengineering Institute, Nanjing, China).

### Western blot analysis

The samples were prepared as previously described [15]. Ipsilateral and contralateral testes were lysed and centrifuged at 4°C (10,000 *g*, 10 minutes). The bicinchoninic acid (BCA) method was used to determine the protein concentration (Pierce, Rockford, IL USA). Equal amounts of protein lysates were subjected to sodium dodecyl sulfate-polyacrylamide (SDS‒PAGE) gel electrophoresis and transferred onto nitrocellulose membranes (Pall Corporation, Pensacola, FL, USA). The immunoblots were incubated overnight at 4°C with primary antibodies against phosphorylated extracellular signal-regulated kinase 1/2 (ERK1/2) (Thr202/Tyr204) (p-ERK) and total ERK1/2 (1:1000, Cell Signaling, Danvers, MA, USA). Then, the membranes were incubated with the secondary anti-rabbit antibody (1:5000, Bio-Rad, Hercules, CA, USA) at room temperature for 1 hour. Signals were detected using a chemiluminescence detection system (Millipore, Billerica, MA, USA) on an Amersham Imager (600, GE Healthcare, Little Chalfont, UK). ImageJ software (National Institutes of Health, Bethesda, MD, USA) was used to analyze the gray value of the band. The densities of phosphorylated bands were normalized to the total protein.

### Immunohistochemical staining

The immunohistochemical staining procedure is described previously [15]. The slices of testicular tissue were subjected to an antigen retrieval procedure according to the manufacturers’ protocol (Beijing Zhongshan Jinqiao Biological Technology Co., Ltd. Beijing, China). Sections were pretreated with H_2_O_2_ prior to blocking. The slices were then incubated with primary antibodies against IL-6, TNF-α, Bcl-2 and Bax (Affinity bioscience, Cincinnati, OH, USA) overnight at 4°C. After that, peroxidase-conjugated goat anti-rabbit secondary antibody (Santa Cruz Biotechnology, Santa Cruz, CA) was used. Next, the slices were developed with 3,3-diaminobenzidine solution (DAB, Beijing Zhongshan Golden Bridge Biotechnology, Beijing, China), and the nuclei were counterstained with hematoxylin. Ten randomly selected images were taken under an upright microscope (Olympus A/S, Ballerup, Denmark) by two researchers blinded to the experimental groups. Image-pro plus software (Media Cybernetics Inc., Carlsbad, CA, USA) was used for analysis.

### Statistics

The results were expressed as the means ± standard deviations (SD). The data analysis was performed using SPSS 26.0 (SPSS Inc., Chicago, IL, USA) and GraphPad Prism 8.0 (GraphPad, La Jolla, CA, USA). Kolmogorov‒Smirnov tests were performed to test the normal distribution of the data. For three groups or more, one-way ANOVA with the Newman–Keuls test or Dunnett’s T3 test was applied for normally distributed data. A value of p<0.05 was considered statistically significant.

## Results

### RIPC protected ipsilateral testes

**Fig 2:**
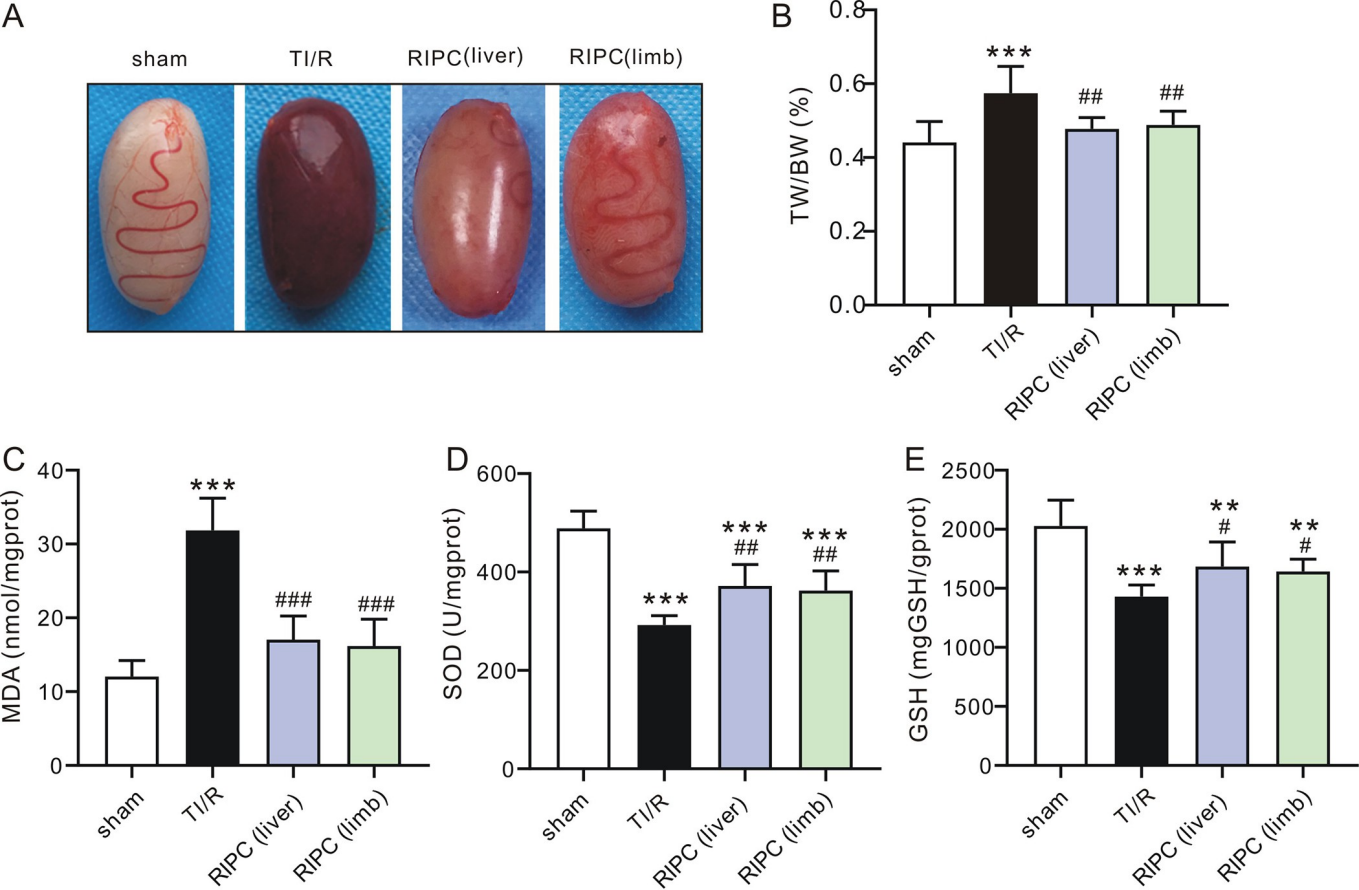
Remote ischemic preconditioning reduces ipsilateral testicular damage after testicular torsion/detorsion injury. **A.** Typical images of ipsilateral testes after TI/R injury. sham: sham-operated; TI/R: testicular ischemia/reperfusion; RIPC (liver): remote liver ischemic preconditioning; RIPC (limb): remote limb ischemic preconditioning. **B.** TW/BW ratio of the ipsilateral testicular tissues postI/R injury. TW: testicular weight; BW: body weight. ***P<0.001, versus sham-operated rats. ^##^P<0.01, versus TI/R. n = 7 per group (by one-way ANOVA). **C.** Changes in ipsilateral testicular MDA levels postdetorsion. MDA: malondialdehyde. ***P<0.001, versus sham-operated rats. ^###^P<0.001, versus TI/R. n = 6 per group (by one-way ANOVA). **D.** Changes in ipsilateral testicular SOD levels postdetorsion. SOD: superoxide dismutase. ***P<0.001, versus sham-operated rats. ^##^P<0.01, versus TI/R. n = 6 per group (by one-way ANOVA). **E.** Changes in ipsilateral testicular GSH levels postdetorsion. GSH: glutathione. **P<0.01, ***P<0.001, versus sham-operated rats. ^#^P<0.05, versus TI/R. n = 6 per group (by one-way ANOVA).

**Fig 3:**
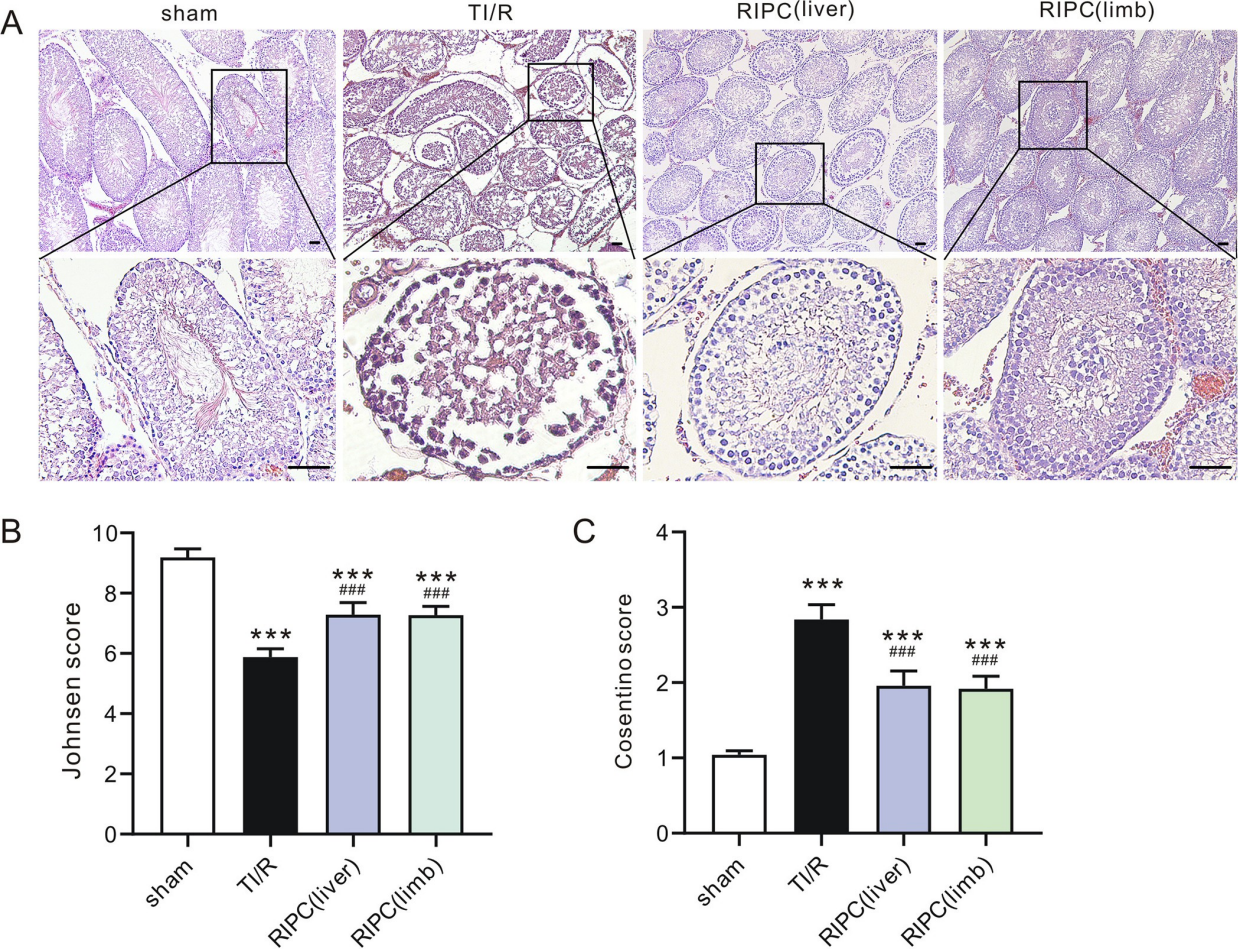
The effect of remote ischemic preconditioning on ipsilateral testicular morphology postI/R. **A.** Typical H&E-stained ipsilateral testicular sections after TI/R injury. sham: sham-operated; TI/R: testicular ischemia/reperfusion; RIPC (liver): remote liver ischemic preconditioning; RIPC (limb): remote limb ischemic preconditioning. Scale bars, 50 μm. n = 5 rats per group. **B.** Johnsen score in the ipsilateral testicular tissues postI/R. ***P<0.001, versus sham; ^###^P<0.001, versus TI/R. n = 5 rats per group (by one-way ANOVA). **C.** Cosentino score in the ipsilateral testicular tissues postI/R. ***P<0.001, versus sham; ^###^P<0.001, versus TI/R. n = 5 rats per group (by one-way ANOVA).

The color of ipsilateral testes isolated from each experimental group is shown in **Fig 2A**. The testes in the TI/R group were dark red. In contrast, RIPC-treated testes were pinkish. Accordingly, testes in the TI/R group exhibited a higher TW/BW ratio (0.57±0.07%) than those in the sham-operated group (0.44±0.06%, P<0.001). However, liver (0.48±0.03%) or limb (0.49±0.04%) ischemic preconditioning-treated testes markedly reduced these swelling responses (all P<0.01 vs. TI/R, **Fig 2B**). Meanwhile, testes in the TI/R group exhibited 1.3-fold higher MDA levels (**Fig 2C**) and 1.7-fold lower SOD activities (**Fig 2D**) than those in the sham-operated group postreperfusion (P<0.001). Both liver and limb ischemic preconditioning were effective at reducing testicular I/R injury, indicated by lower testicular MDA levels and higher SOD activities compared with those in the TI/R group (P<0.01, or P<0.001). In addition, GSH levels were decreased in the TI/R group after detachment (P<0.001 vs. sham), and rats in the RIPC group had a significant increase in GSH levels compared with those in the TI/R group (P<0.05, **Fig 2E**). In addition, testicular torsion/detorsion caused significant ipsilateral testicular damage, i.e., testicular edema, congestion, hemorrhage, necrosis. In contrast, rats that received RIPC treatment exhibited less severe testicular histological alterations (**Fig 3A**). Two scoring systems were used to evaluate the pathological profile. The Johnsen score was adopted for spermatogenesis assessment [14], and the Cosentino score was used for quantitation of histological changes in seminiferous tubules [13]. As shown in **Fig 3B and 3C**, testicular torsion/detorsion led to a decreased Johnsen score (5.9±0.3) and an increased Cosentino score (2.8±0.2), suggesting testis and spermatogenesis damage. However, liver (Johnsen: 7.3±0.4, Cosentino: 2.0±0.2) or limb (Johnsen: 7.3±0.3, Cosentino: 2.0±0.2) preconditioning ameliorated these lesions, as seen by a more remarkable increase in the Johnsen score and decrease in the Cosentino score when compared with TI/R (all P<0.001).

**Fig 4:**
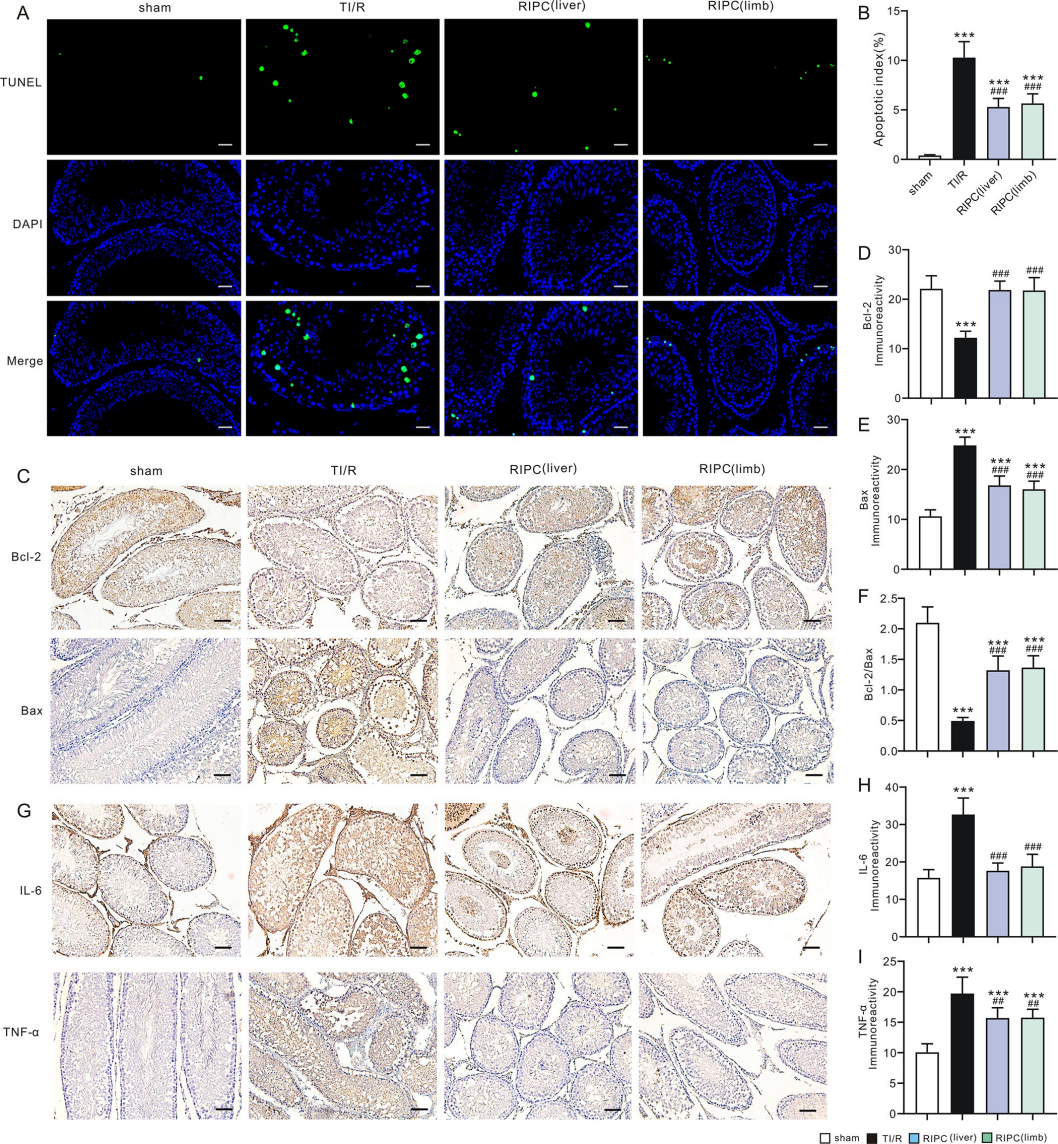
Remote ischemic preconditioning ameliorates ipsilateral testicular apoptosis and the inflammatory response postI/R. **A.** TUNEL-stained ipsilateral testicular sections. Green: TUNEL-positive nuclei; Blue: DAPI-stained total nuclei. sham: sham-operated; TI/R: testicular ischemia/reperfusion; RIPC (liver): remote liver ischemic preconditioning; RIPC (limb): remote limb ischemic preconditioning. Scale bars, 20 μm. n = 5 rats per group. **B.** Apoptotic index (TUNEL-positive neclei*100%/total nuclei). n = 5 per group. ***P<0.001, versus sham; ^###^P<0.001, versus TI/R (by one-way ANOVA). **C.** The immunostaining for Bcl-2 and Bax in ipsilateral testicular sections after detorsion. Scale bars, 50 μm. **D.** The level of immunoreactivity of Bcl-2. n = 5 per group. ***P<0.001, versus sham; ^###^P<0.001, versus TI/R (by one-way ANOVA). **E.** The level of immunoreactivity of Bax. n = 5 per group. ***P<0.001, versus sham; ^###^P<0.001, versus TI/R (by one-way ANOVA). **F.** Bcl-2/Bax ratio. n = 5 per group. ***P<0.001, versus sham; ^###^P<0.001, versus TI/R (by one-way ANOVA). **G.** The immunostaining for IL-6 and TNF-α in ipsilateral testicular sections after detorsion. Scale bars, 50 μm. **H.** Testicular immunoreactivity levels of IL-6 protein. n = 5 per group. ***P<0.001, versus sham; ^###^P<0.001, versus TI/R (by one-way ANOVA). **I.** Testicular immunoreactivity levels of TNF-α protein. n = 5 per group. ***P<0.001, versus sham; ^##^P<0.01, versus TI/R (by one-way ANOVA).

Using the TUNEL staining technique, we evaluated ipsilateral testicular apoptosis postI/R and found that the apoptotic index was decreased in liver (5.3±0.9%) and limb (5.7±1.0%)-preconditioning-treated rats compared with TI/R rats (10.3±1.6, P<0.001, **Fig 4A and 4B**). Furthermore, the expression of Bcl-2 and Bax in the testicular sections 3 h after reperfusion was shown by immunohistochemistry staining (**Fig 4C**). The expression levels of antiapoptotic proteins such as Bcl-2 were found to be lower in TI/R rats than in sham rats, whereas the proapoptotic protein BAX was more highly distributed in TI/R-treated testes than in sham-operated rats. In contrast, RIPC treatment significantly ameliorated apoptosis, as evidenced by increased ipsilateral testicular expression of Bcl-2 protein (P<0.001, **Fig 4D**) and reduced Bax expression (P<0.001, **Fig 4E**), resulting in a higher Bcl-2-to-Bax ratio when compared with TI/R (P<0.001, **Fig 4F**).

We also examined the expression levels of the proinflammatory cytokines interleukin-6 and tumor necrosis factor-α in ipsilateral testes by immunohistochemistry staining (**Fig 4G**). As shown in **Fig 4H and 4I**, IL-6 and TNF-α were massively expressed in TI/R-treated testes (P<0.001 vs. sham). However, RIPC treatment markedly reduced the expression levels of IL-6 and TNF-α protein when compared with TI/R testes (P<0.01 or P<0.001), suggesting that RIPC could ameliorate ipsilateral testicular inflammatory responses after testicular I/R.

### RIPC ameliorated contralateral testicular damage

**Fig 5:**
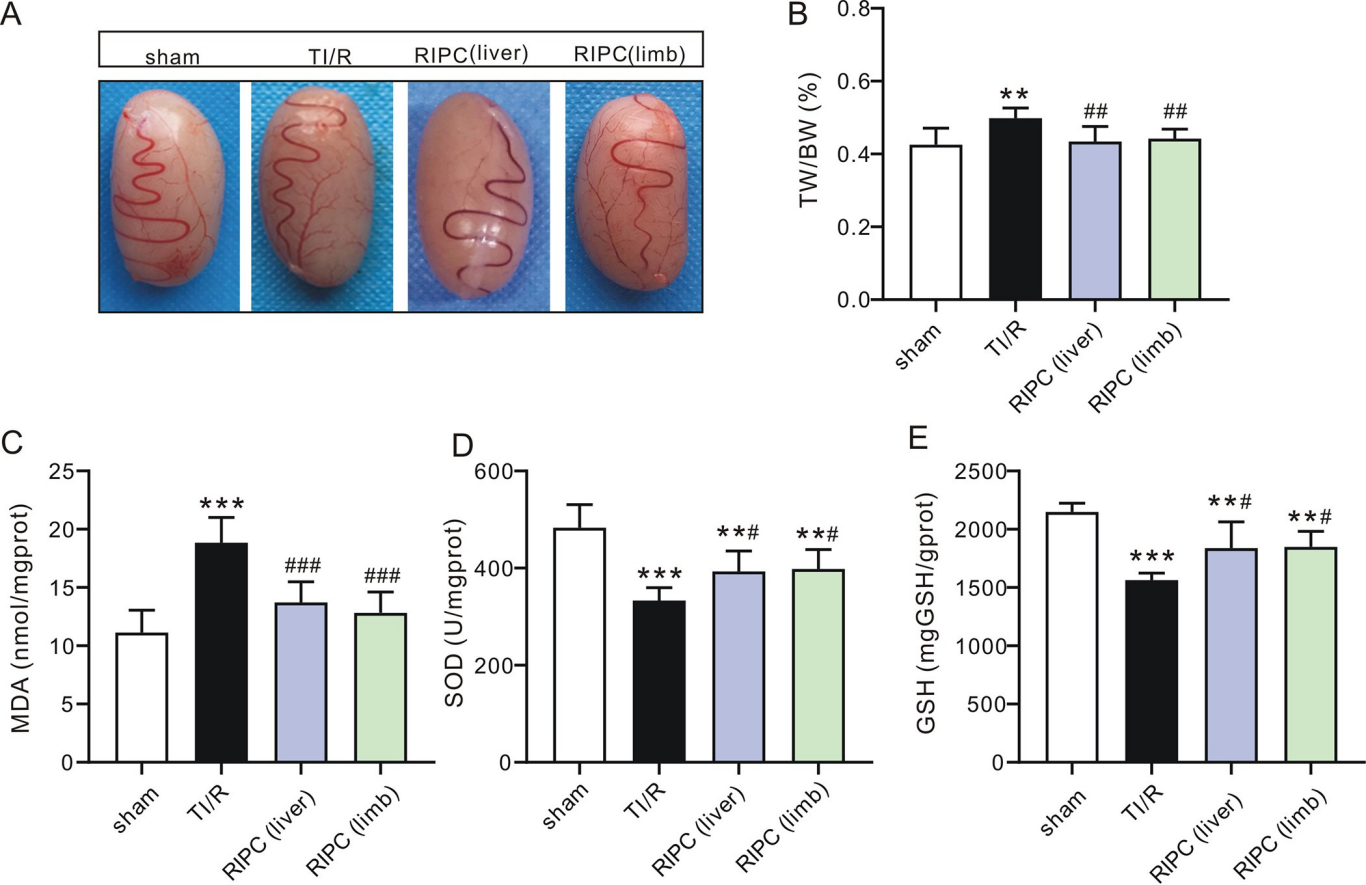
Remote ischemic preconditioning reduces contralateral testicular damage after testicular torsion/detorsion injury. **A.** Typical images of contralateral testes after TI/R injury. sham: sham-operated; TI/R: testicular ischemia/reperfusion; RIPC (liver): remote liver ischemic preconditioning; RIPC (limb): remote limb ischemic preconditioning. **B.** TW/BW ratio of the contralateral testicular tissues postI/R injury. TW: testicular weight; BW: body weight. **P<0.01, versus sham-operated rats. ^##^P<0.01, versus TI/R. n = 7 per group (by one-way ANOVA). **C.** Changes in contralateral testicular MDA levels postdetorsion. MDA: malondialdehyde. ***P<0.001, versus sham-operated rats. ^###^P<0.001, versus TI/R n = 6 per group (by one-way ANOVA). **D.** Changes in contralateral testicular SOD levels postdetorsion. SOD: superoxide dismutase. **P<0.01, ***P<0.001, versus sham-operated rats. ^#^P<0.05, versus TI/R. n = 6 per group (by one-way ANOVA). **E.** Changes in contralateral testicular GSH levels postdetorsion. GSH: glutathione. **P<0.01, ***P<0.001, versus sham-operated rats. ^#^P<0.05, versus TI/R. n = 6 per group (by one-way ANOVA).

We found that RIPC protected ipsilateral testes after testicular torsion/detorsion. We further examined whether these beneficial effects were also observed in the contralateral testes. The color of the contralateral testes is shown in **Fig 5A**. It seems that testicular torsion occurring in the ipsilateral testis did not significantly affect the appearance of the contralateral testis. However, we observed that ipsilateral testicular I/R injury could also lead to significant contralateral testicular damage; for example, the ratio of contralateral TW/BW was more than 1.2-fold greater in the TI/R group (0.50±0.03%) than in the sham group (0.43±0.05%, P<0.01). However, treatment with RIPC significantly reduced testicular weight-to-body weight (P<0.01 vs. TI/R, **Fig 5B**). Additionally, the contralateral testes showed higher MDA levels, accompanied by lower SOD activities after TI/R when compared with sham testes (P<0.01 or P<0.001). GSH levels were decreased in contralateral testes after ipsilateral testicular I/R (P<0.001 vs. sham). Consistent with the findings in ipsilateral testes, we also observed that RIPC treatment significantly alleviated this oxidative damage in contralateral testes (all P<0.05 or P<0.001 vs. TI/R, **Fig 5C–5E**).

**Fig 6:**
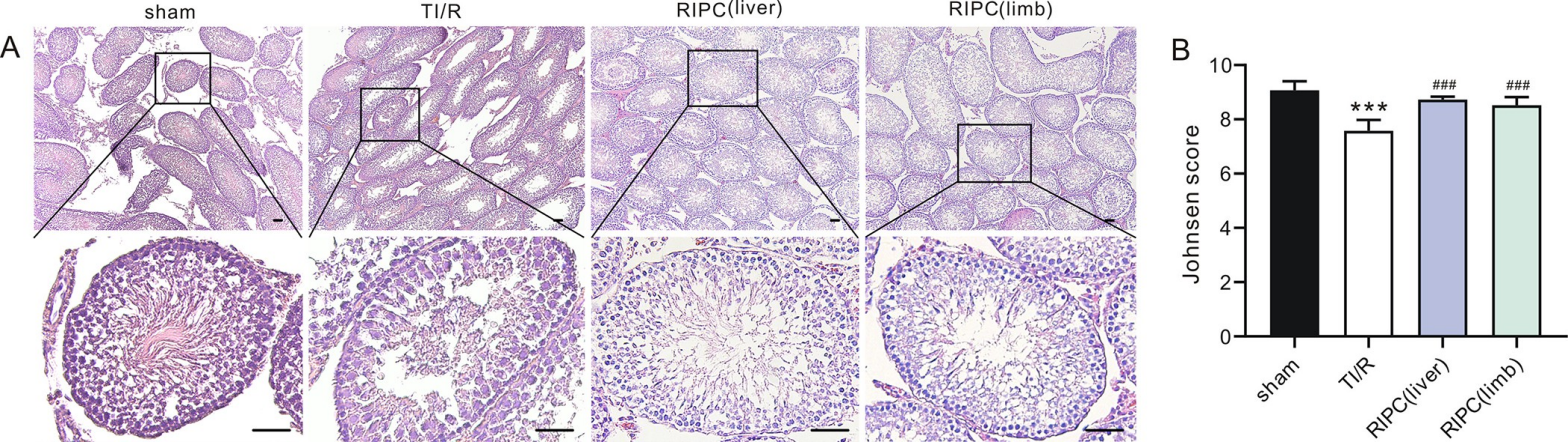
The effect of remote ischemic preconditioning on contralateral testicular morphology postI/R. **A.** Typical H&E-stained contralateral testicular sections after TI/R injury. sham: sham-operated; TI/R: testicular ischemia/reperfusion; RIPC (liver): remote liver ischemic preconditioning; RIPC (limb): remote limb ischemic preconditioning. Scale bars, 50 μm. n = 5 rats per group. **B.** Johnsen score in the contralateral testicular tissues postI/R. ***P<0.001, versus sham; ^###^P<0.001, versus TI/R. n = 5 rats per group (by one-way ANOVA).

**Fig 7:**
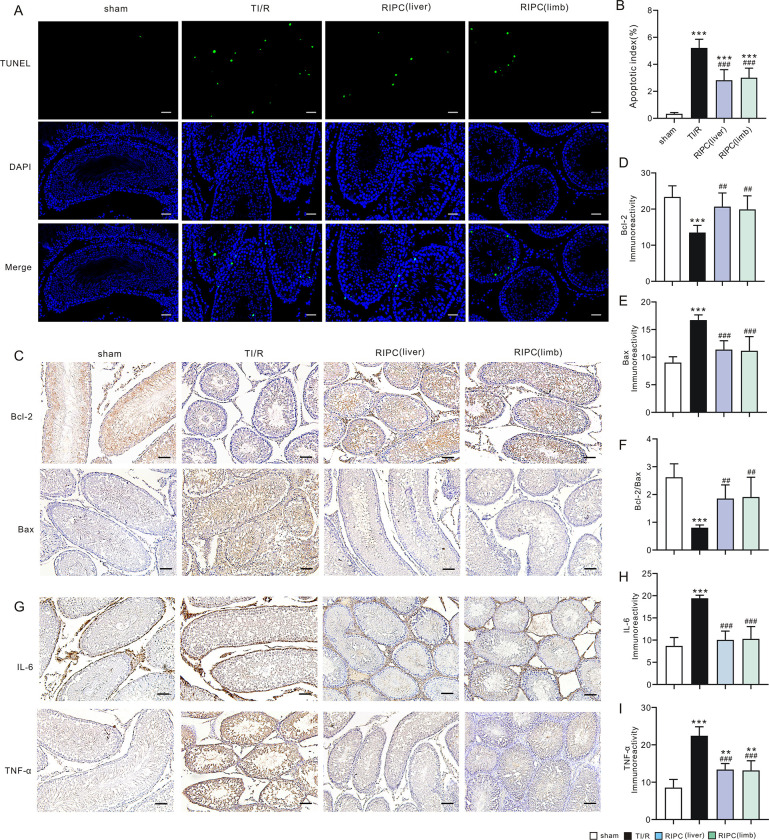
Remote ischemic preconditioning ameliorates contralateral testicular apoptosis and the inflammatory response postI/R. **A.** TUNEL-stained contralateral testicular sections. Green: TUNEL-positive nuclei; Blue: DAPI-stained total nuclei. sham: sham-operated; TI/R: testicular ischemia/reperfusion; RIPC (liver): remote liver ischemic preconditioning; RIPC (limb): remote limb ischemic preconditioning. Scale bars, 20 μm. n = 5 rats per group. **B.** Apoptotic index (TUNEL-positive neclei*100%/total nuclei). n = 5 per group. ***P<0.001, versus sham; ^###^P<0.001, versus TI/R (by one-way ANOVA). **C.** The immunostaining for Bcl-2 and Bax in contralateral testicular sections after detorsion. Scale bars, 50 μm. **D.** The level of immunoreactivity of Bcl-2. n = 5 per group. ***P<0.001, versus sham; ^##^P<0.01, versus TI/R (by one-way ANOVA). **E.** The level of immunoreactivity of Bax. n = 5 per group. ***P<0.001, versus sham; ^###^P<0.001, versus TI/R (by one-way ANOVA). **F.** Bcl-2/Bax ratio. n = 5 per group. ***P<0.001, versus sham; ^##^P<0.01, versus TI/R. **G.** The immunostaining for IL-6 and TNF-α in contralateral testicular sections after detorsion. Scale bars, 50 μm. **H.** Testicular immunoreactivity levels of IL-6 protein. n = 5 per group. ***P<0.001, versus sham; ^###^P<0.001, versus TI/R (by one-way ANOVA). **I.** Testicular immunoreactivity levels of TNF-α protein. n = 5 per group. **P<0.01, ***P<0.001, versus sham; ^##^P<0.01, versus TI/R (by one-way ANOVA).

H&E staining revealed that sham-operated rats had normal morphology, while contralateral testes had impaired spermatogenesis and presented disorganized tubules. Liver (Johnsen score: 8.7±0.1) or limb (Johnsen score: 8.5±0.3) preconditioning treatment caused few testicular histological alterations in contralateral testes when compared with TI/R rats (Johnsen score: 7.6±0.4, all P<0.001, **Fig 6A and 6B**). Furthermore, we found that liver (2.8±0.8) and limb (3.0±0.7) preconditioning inhibited contralateral testicular apoptosis, as seen by a reduced apoptotic index when compared with TI/R rats (5.2±0.6, all P<0.001, **Fig 7A and 7B**). Consistent with this, RIPC also increased Bcl-2 (anti-apoptotic protein) expression and inhibited Bax expression (pro-apoptotic protein) in contralateral testes (P<0.01 or P<0.001 vs. TI/R, **Fig 7C–7F**). Similar to our findings in the ipsilateral testes, RIPC could reduce the expression levels of IL-6 and TNF-α in contralateral testes (P<0.001 vs. TI/R, **Fig 7G–7I**), indicating that RIPC was also effective in ameliorating contralateral testicular inflammatory responses.

### RIPC enhanced testicular ERK1/2 phosphorylation

**Fig 8:**
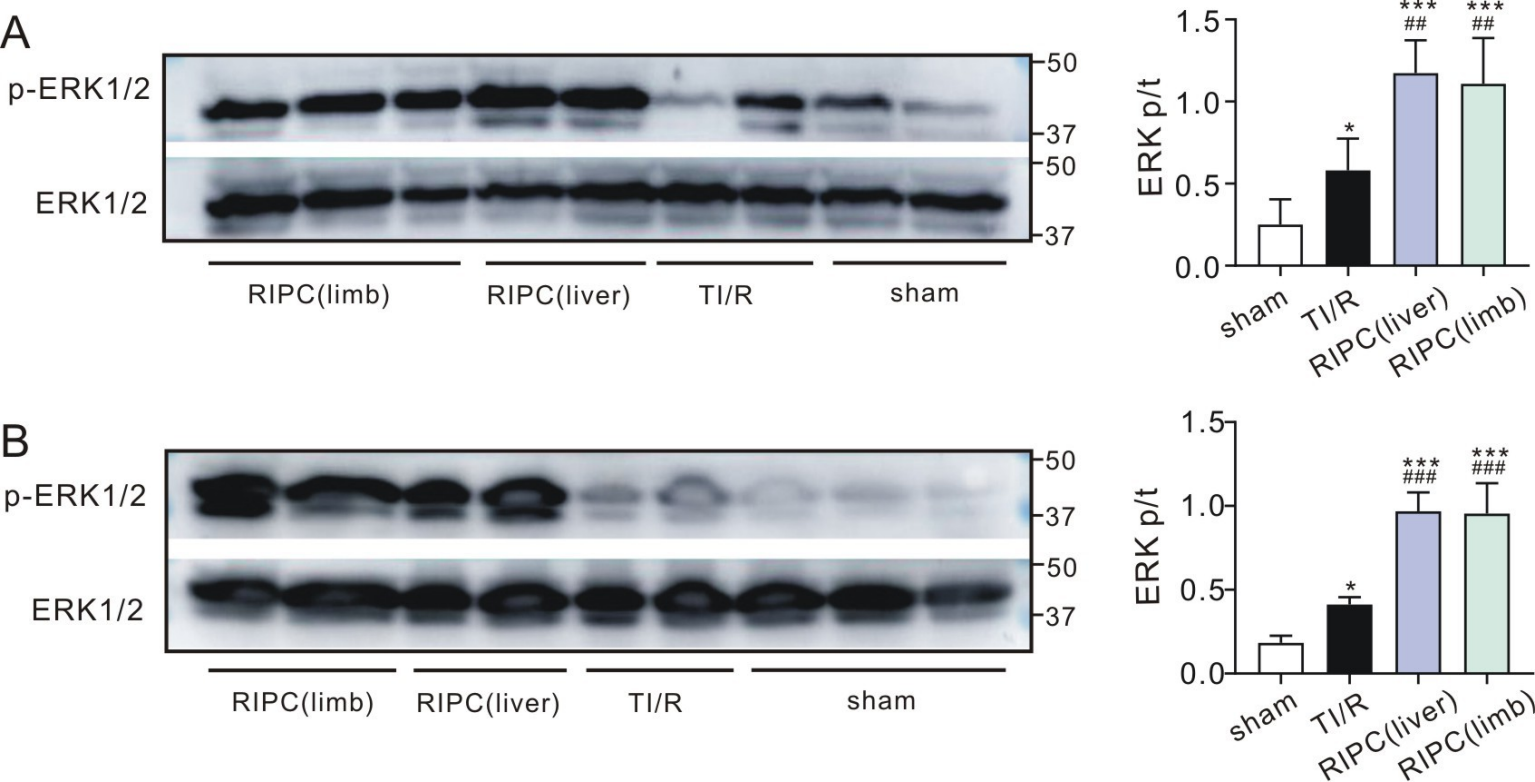
Remote ischemic preconditioning enhances testicular ERK1/2 phosphorylation. ***A*. *Left***, western blots of ipsilateral testicular phosphorylated ERK1/2 and total ERK1/2. ***Right*,** mean band densities of p-ERK1/2/ERK1/2. n = 4 per group. *P<0.05, ***P<0.001, versus sham; ^##^P<0.01, versus TI/R (by one-way ANOVA). ***B*. *Left***, western blots of contralateral testicular phosphorylated ERK1/2 and total ERK1/2. ***Right*,** mean band densities of p-ERK1/2/ERK1/2. n = 4 per group. *P<0.05, ***P<0.001, versus sham; ^###^P<0.001, versus TI/R (by one-way ANOVA).

We found that the ratios of ipsilateral testicular phosphor-ERK1/2 to total ERK1/2 were increased upon TI/R treatment compared with sham-operated rats (P<0.05). However, the phosphorylation levels of ERK1/2 in ipsilateral testes were increased by 2.0- and 1.9-fold in the liver and limb preconditioning groups, respectively, compared with the TI/R group (all, P<0.01, **Fig 8A**). Meanwhile, we also found that testicular detorsion activated ERK1/2 in the contralateral testes, resulting in an increase in the phosphor-ERK1/2 to total ERK1/2 ratio (P<0.05 vs. sham). Furthermore, the phosphorylation level of ERK1/2 was increased by more than 2-fold upon RIPC treatment (P<0.001 vs. TI/R, **Fig 8B**). These results indicated that RIPC was capable of enhancing both ipsilateral and contralateral testicular ERK1/2 phosphorylation post testicular I/R.

### U0126 abolished RIPC-mediated testicular protection

**Fig 9:**
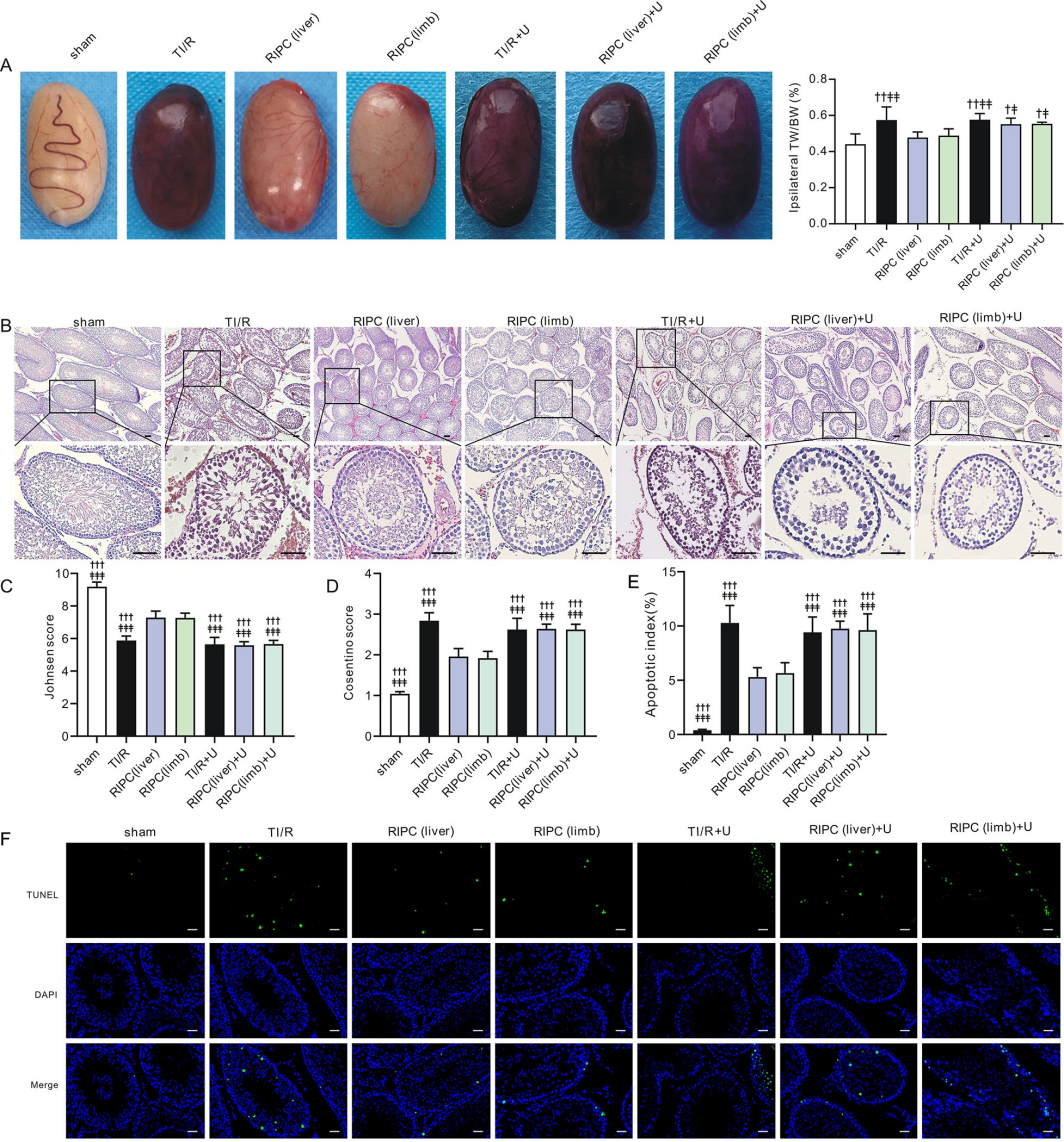
Pharmacological inhibition of ERK1/2 abolishes RIPC-induced ipsilateral testicular protection. **A. *Left*,** Typical images of ipsilateral testes after TI/R injury. sham: sham-operated; TI/R: testicular ischemia/reperfusion; RIPC (liver): remote liver ischemic preconditioning; RIPC (limb): remote limb ischemic preconditioning. U: U0126. ***Right*,** TW/BW ratio of the ipsilateral testicular tissues postI/R injury. TW: testicular weight; BW: body weight. ^†^P<0.05, ^††^P<0.01, versus RIPC (liver) rats. ^‡^P<0.05, ^‡‡^P<0.01, versus RIPC (limb) rats. n = 6–7 per group. Values for sham, TI/R and RIPC rats are repeated from Fig 2B for comparison (by one-way ANOVA). **B.** Typical H&E-stained ipsilateral testicular sections after TI/R injury. Scale bars, 50 μm. n = 5 rats per group. **C.** Johnsen score in the ipsilateral testicular tissues postI/R. n = 5 rats per group. ^†††^P<0.001, versus RIPC (liver) rats. ^‡‡‡^P<0.001, versus RIPC (limb) rats. n = 5 per group. Values for sham, TI/R and RIPC rats are repeated from Fig 3B for comparison (by one-way ANOVA). **D**. Consentino score in the ipsilateral testicular tissues postI/R. n = 5 rats per group. ^†††^P<0.001, versus RIPC (liver) rats. ^‡‡‡^P<0.001, versus RIPC (limb) rats. n = 5 per group. Values for sham, TI/R and RIPC rats are repeated from Fig 3C for comparison (by one-way ANOVA). **E**. Apoptotic index (TUNEL-positive nuclei*100%/total nuclei). n = 5 per group. ^†††^P<0.001, versus RIPC (liver) rats. ^‡‡‡^P<0.001, versus RIPC (limb) rats. Values for sham, TI/R and RIPC rats are repeated from Fig 4B for comparison (by one-way ANOVA). **F**. TUNEL-stained testicular sections. Scale bars, 20 μm. n = 5 rats per group.

We showed that RIPC could enhance ERK1/2 phosphorylation in both ipsilateral and contralateral testes after ipsilateral testicular detorsion. To further evaluate the role of ERK1/2 in RIPC-induced testicular protection, U0126, a MAPK/ERK kinase (MEK)-specific inhibitor, was used in the study. We found that the beneficial effect of RIPC was abolished after U0126 application. Typical images of ipsilateral testes are shown in **Fig 9A**. Remote liver or limb ischemic preconditioning-treated testes became dark in color after U0126 treatment. Consistent with this, the ratio of ipsilateral TW/BW was elevated in the RIPC(liver)+U0126 and RIPC(limb)+U0126 groups after detorsion and was equivalent to the levels in TI/R rats (P<0.05 or P<0.01 vs. RIPC without U0126, **Fig 9A**). As shown in **Fig 9B**, the degree of testicular injury was much more severe in RIPC+U0126 rats than in RIPC rats.

Rats in the RIPC(liver)+U0126 (5.6±0.2) or RIPC(limb)+U0126 (5.7±0.2) group had higher Johnson scores than those in the RIPC(liver) (7.3±0.4) or RIPC(limb) (7.3±0.3) group (all, P<0.001, **Fig 9C**). Similarly, the Cosentino values were much higher in the RIPC groups with U0126 than in the RIPC group in the absence of U0126 (all, P<0.001, **Fig 9D**). In addition, TUNEL-positive stained nuclei were observed in the ipsilateral testes postI/R. RIPC-treated testes with U0126 exhibited increased numbers of testicular apoptotic nuclei when compared with RIPC-treated testes in the absence of inhibitor (all, P<0.001, **Fig 9E and 9F**).

**Fig 10:**
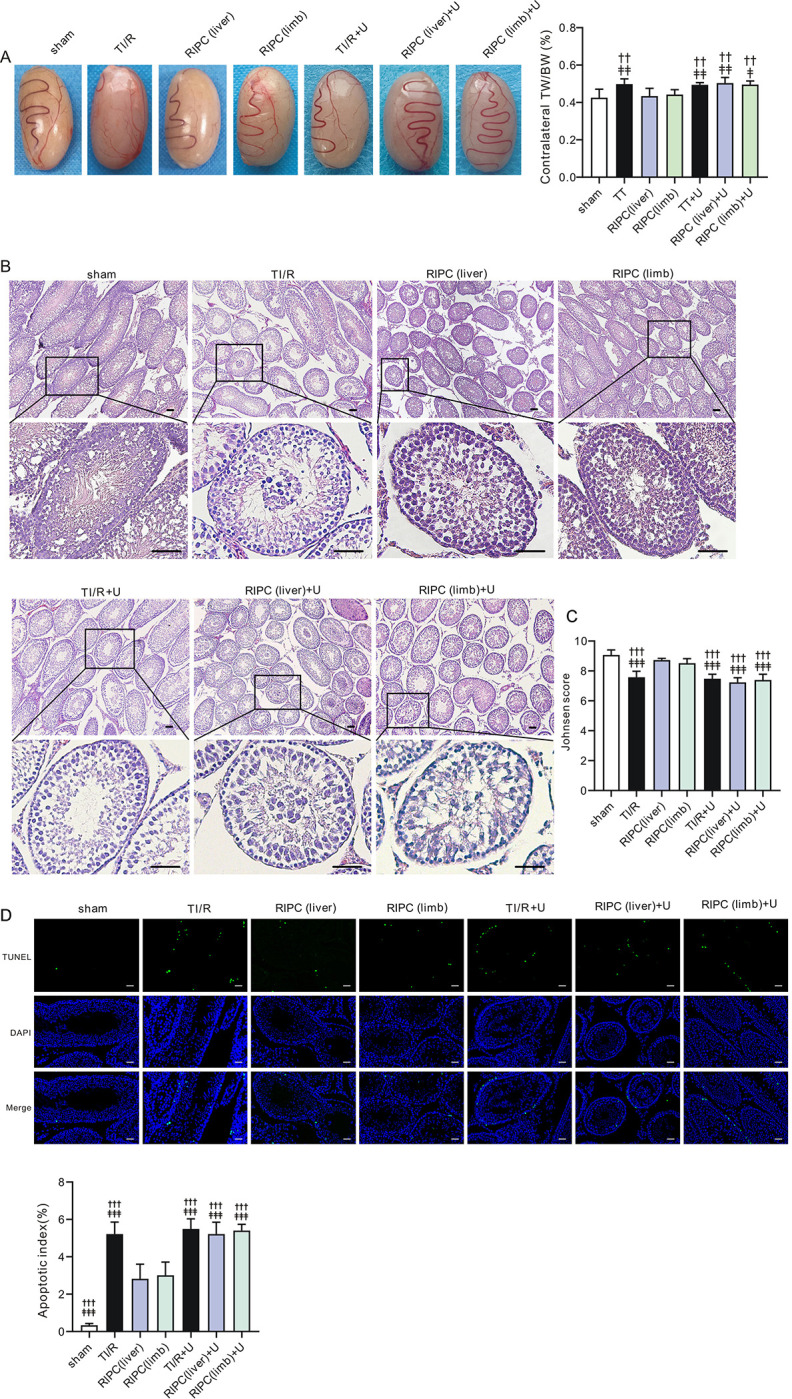
Pharmacological inhibition of ERK1/2 abolishes RIPC-induced contralateral testicular protection. A. *Left*, Typical images of contralateral testes after TI/R injury. sham: sham-operated; TI/R: testicular ischemia/reperfusion; RIPC (liver): remote liver ischemic preconditioning; RIPC (limb): remote limb ischemic preconditioning. U: U0126. ***Right*,** TW/BW ratio of the contralateral testicular tissues postI/R injury. TW: testicular weight; BW: body weight. ^††^P<0.01, versus RIPC (liver) rats. ^‡^P<0.05, ^‡‡^P<0.01, versus RIPC (limb) rats. n = 6–7 per group. Values for sham, TI/R and RIPC rats are repeated from Fig 5B for comparison (by one-way ANOVA). **B.** Typical H&E-stained contralateral testicular sections after TI/R injury. Scale bars, 50 μm. n = 5 rats per group. **C.** Johnsen score in the contralateral testicular tissues postI/R. n = 5 rats per group. ^†††^P<0.001, versus RIPC (liver) rats. ^‡‡‡^P<0.001, versus RIPC (limb) rats. n = 5 per group. Values for sham, TI/R and RIPC rats are repeated from Fig 6B for comparison (by one-way ANOVA). **D.** Typical TUNEL-stained contralateral testicular sections (***upper***) and apoptotic index (TUNEL-positive nuclei*100%/total nuclei, ***lower***). n = 5 per group. ^†††^P<0.001, versus RIPC (liver) rats. ^‡‡‡^P<0.001, versus RIPC (limb) rats. Values for sham, TI/R and RIPC rats are repeated from Fig 7B for comparison (by one-way ANOVA). Scale bars, 20 μm.

Similar findings were observed in contralateral testes. Although it seems that testicular appearances were not affected by the application of U0126, the protective effect offered by RIPC on contralateral testes was abolished by U0126, as seen by the increase in the TW/BW ratio in RIPC-treated testes with U0126 (P<0.05 or P<0.01 vs. RIPC without U0126, **Fig 10A**). H&E staining showed that U0126 also deteriorated the contralateral testicular structure, as evidenced by decreased Johnsen scores in the RIPC+U0126 groups compared with the RIPC groups (all, P<0.001, **Fig 10B and 10C**). Additionally, the mean apoptotic index was 1.84-fold and 1.80-fold higher, respectively, in RIPC(liver)+U0126 (5.2±0.6, P<0.001) and RIPC(limb)+U0126 (5.4±0.3, P<0.001) rats than in RIPC(liver) (2.8±0.8) and RIPC(limb)(3.0±0.7) rats, suggesting that U0126 abolished the RIPC-mediated anti-apoptotic effect in contralateral testes after testicular I/R injury (**Fig 10D**).

### RIPC-induced protection is mediated by ERK1/2 phosphorylation

**Fig 11:**
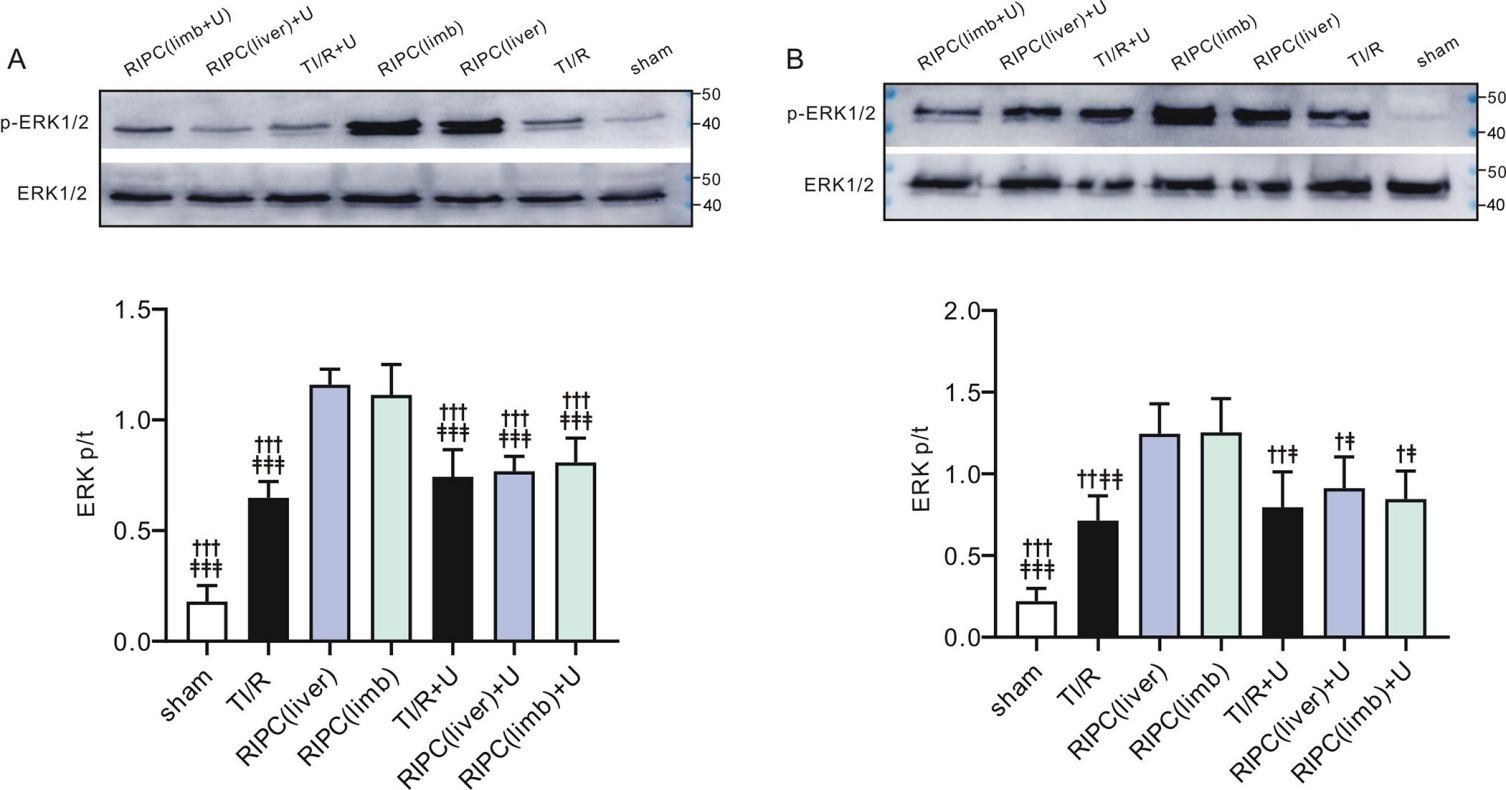
U0126 diminishes RIPC-induced testicular ERK1/2 phosphorylation. ***A*.** Western blots (***upper***) and band densities (***lower***) of p-ERK1/2/ERK1/2 in rat ipsilateral testes with or without U0126 (U). n = 4 per group. ^†††^P<0.001, versus RIPC (liver) rats. ^‡‡‡^P<0.001, versus RIPC (limb) rats (by one-way ANOVA). ***B*.** Western blots (***upper***) and band densities (***lower***) of p-ERK1/2/ERK1/2 in rat contralateral testes with or without U0126. n = 4 per group. ^†^P<0.05, ^††^P<0.01, ^†††^P<0.001, versus RIPC (liver) rats. ^‡^P<0.05, ^‡‡^P<0.01,^‡‡‡^P<0.001, versus RIPC (limb) rats (by one-way ANOVA).

We then evaluated the phosphorylation level of ERK1/2 in both ipsilateral and contralateral testes after U0126 application. We found that U0126 significantly blocked RIPC-induced ipsilateral testicular ERK1/2 phosphorylation when compared with the non-U0126-treated RIPC group (P<0.001, **Fig 11A**). Similarly, U0126 also caused a significant decrease in ERK1/2 phosphorylation in contralateral testes when compared with rats with RIPC only, to a level similar to that of testes in the TI/R group (P<0.05, **Fig 11B**).

## Discussion

We showed in our study that remote ischemic preconditioning attenuated both ipsilateral and contralateral testicular damage after testicular I/R injury. Specifically, RIPC effectively reduced testicular swelling, alleviated structural abnormalities and testicular oxidative stress, and inhibited testicular apoptosis and the inflammatory response. Meanwhile, this RIPC-induced testicular protection may be associated with the activation of the ERK1/2 signaling pathway.

Ischemic preconditioning is a classic endogenous protective therapy. Murry et al. first applied a short period of I/R before an extended ischemic insult and found that the infarct size was largely reduced [2]. IPC has been tested in most organs and animal models and has been repeatedly shown to reduce organ damage after I/R injury [16–18]. Accordingly, IPC was found to protect the testis against testicular I/R injury [3]. Furthermore, the beneficial effect of IPC has also been confirmed in clinical settings [19]. In 1993, the concept of remote ischemic preconditioning (RIPC) was introduced by Przyklenk et al. RIPC requires only brief interruption of vascular blood flow in the remote site and can effectively protect against subsequent prolonged ischemia or reperfusion injury in the target organ. They showed that four cycles of 5 minutes of circumflex branch ischemia and reperfusion stimuli performed prior to 1 hour of left anterior descending coronary artery occlusion resulted in limited infarction in a canine model [4]. Later, in 1997, Birnbaum et al. showed that an infarct-sparing effect can be achieved by transient limb ischemia before coronary artery occlusion [20]. RIPC is a cost-efficient, clinically feasible and easy procedure and thus serves as a successful strategy in providing organ salvage against I/R injury. Similar to IPC, the efficiency of RIPC has been convincingly confirmed in humans and numerous species of animals worldwide [21]. However, to the best of our knowledge, there are no studies reporting associations between RIPC and testis protection in terms of testicular I/R injury. We found for the first time that remote ischemic preconditioning executed on the limb or internal organ (liver) before testicular torsion could successfully produce strong protection to both ipsilateral and contralateral testes after detorsion *in vivo*. Our current observations may shed light on the protective role of RIPC against testicular I/R injury. In agreement with our findings, Mansour et al. have demonstrated that tail clamping during testicular torsion period (before testicular detorsion) could exert testicular protection [22].

Free radical damage (mainly oxidative damage) has been shown to play a key role in the pathophysiological process of I/R injury. The formation of free radicals is boosted after I/R and subsequently triggers cellular injury [23]. Malondialdehyde (MDA) is a final product of lipid peroxidation and is a marker of oxidative damage. I/R injury causes overproduction of MDA. Superoxide dismutase (SOD) is a metalloenzyme that serves as a radical superoxide scavenger and defense against oxidative stress. I/R injury causes lower SOD activity. Meanwhile, another major endogenous antioxidant, glutathione (GSH), can be depleted upon I/R injury [24]. In our study, we found that testicular MDA levels were significantly increased, accompanied by impaired SOD activity and GSH depletion in the testicular I/R group in both ipsilateral and contralateral testes when compared with the sham group, indicative of oxidative damage postI/R. Our results are in line with others showing that testes are sensitive to free radical damage [25, 26]. However, RIPC treatment effectively ameliorated oxidative damage and decreased oxidant generation after testicular I/R by reducing MDA levels, increasing the activities of SOD and ameliorating GSH depletion, suggesting an important mechanism contributing to RIPC-mediated testicular protection.

A number of preclinical and clinical studies suggest that unilateral testicular torsion/detorsion also affects the contralateral testis, as seen by impaired testicular and spermatogenic function [6, 27]. The exact mechanism responsible for contralateral testicular deterioration is not clear. The proposed theories include the immune response, increased oxidative stress, or hypoxia [28, 29]. Our study extends these findings by showing that three hours of unilateral testicular torsion followed by 3 h of detorsion affected contralateral testes, as evidenced by increased oxidative damage, inflammatory response, apoptosis, and impaired morphological structure. Here, we further showed that RIPC not only protected ipsilateral testes but also effectively ameliorated contralateral testicular damage. In contrast, it has been reported that remote ischemic conditioning performed during testicular ischemia (remote ischemic perconditioning) failed to protect contralateral testes [22]. The discrepancy may result from differences in the ischemic conditioning strategies, the site of conditioning application, and the experimental design.

Apoptosis plays an important role in the pathophysiology of ischemia and reperfusion injury [30]. The restoration of blood flow during the detorsion period may trigger and cause massive apoptosis in spermatogenic cells, thus inducing progressive damage to spermatogenesis and testicular dysfunction [25]. Apoptosis is highly regulated by the antiapoptotic Bcl-2 and proapoptotic Bax proteins. The ratio of Bcl-2 to Bax determines cell survival or death [31]. Accordingly, we found in the current study that I/R-treated ipsilateral and contralateral testes had more apoptotic nuclei than sham testes. However, remote preconditioning significantly inhibited testicular apoptosis, accompanied by an increase in Bcl-2 protein expression and a decrease in Bax protein expression, thus resulting in a greater Bcl-2-to-Bax ratio than that of the non-RLIPC-treated TI/R group. I/R injury also stimulates the inflammatory response [32]. TNF-α and IL-6 are essential components of the postreperfusion inflammatory cascade [33]. We therefore tested these two important inflammatory mediators in the current study. It is well accepted that their expression levels can be increased upon I/R injury [34]. We showed that both ipsilateral and contralateral testes had elevated expression levels of TNF-α and IL-6 after testicular I/R injury. In contrast, RIPC significantly decreased their expression, revealing that RIPC can regulate the inflammatory response in response to testicular I/R injury.

Extracellular signal-regulated kinase (ERK1/2), a serine/threonine protein kinase, is one of the most important members of the mitogen-activated protein kinase (MAPK) family. ERK1/2 regulates a wide range of physiological processes, including inflammation, metabolism, cell death and survival, cell cycle progression, transcription, and cell cycle progression [35]. ERK1/2 has been shown to play a significant role in I/R injury. Activation of the ERK1/2 pathway (via phosphorylation) occurs in response to stress stimuli, such as hypoxia, inflammation, or I/R injury. It has been shown that protective interventions (ischemic conditioning) or agents (volatile anesthetics) can cause ERK1/2 activation [36, 37]. In contrast, ischemic conditioning- or drug-induced cardioprotection can be abolished by pharmacological inhibition of ERK1/2 activation [8, 15]. The current results also confirm these previous findings demonstrating that liver preconditioning and limb preconditioning both increased ERK1/2 phosphorylation in both ipsilateral and contralateral testes after testicular I/R injury, suggesting that ipsilateral and contralateral testes may share similar mechanisms in response to RIPC-induced testicular protection. Additionally, we found that inhibition of ERK1/2 phosphorylation by an ERK1/2 inhibitor abolished the beneficial effect offered by RIPC. The underlying mechanism for the beneficial effects of remote preconditioning and postconditioning (RIPost) is not completely understood and has been attributed to the modulation of signaling pathways [38]. Studies have shown that RIPC and RIPost, two endogenous cardioprotective phenomena, may activate similar pathways and signal transduction cascades in the heart [39]. It is worth mentioning that we previously found that remote ischemic conditioning executed after testicular detorsion (RIPost) protected rat testes against testicular I/R injury. However, ERK1/2 phosphorylation was increased only in RIPost-treated ipsilateral testes but not in contralateral testes after detorsion when compared to rats in the non-RIPost-treated testicular I/R group [12]. Taken together, our results indicate that RIPC and RIPost may protect ipsilateral testis against testicular I/R via activation of ERK1/2. Nevertheless, the possible mechanism underlying RIPC and RIPost-mediated testicular protection may be different in contralateral testes.

We acknowledge several limitations of this study. First, we used two remote ischemic preconditioning protocols (RIPC limb and RIPC liver), and whether these protective ischemia‒reperfusion procedures applied in other distant tissues or vascular beds may exert testicular protection remains unknown. Meanwhile, we used four cycles of a brief I/R protocol prior to testicular torsion; thus, it is unclear whether other stimuli protocols would be protective and ameliorate testicular damage. Second, we found that ERK1/2, a MAPK family member, was activated after RIPC treatment. Although ERK1/2 may be a candidate molecule linked with RIPC-associated testicular protection, large genetic or proteome screening and verification may be needed in the future. In addition, the mechanisms by which RIPC protects the testis seem to be associated with the MAPK signaling pathway. Therefore, it is worth exploring the roles of other members of the MAPK signaling cascades in the future. Third, in the current study, we did not detect any difference regarding the therapeutic efficiency of limb preconditioning and liver preconditioning. However, they may act differently when the experimental protocol changes.

In conclusion, the present study demonstrates that remote ischemic preconditioning protects both ipsilateral and contralateral testes against testicular I/R injury, which may occur as a consequence of testicular ERK1/2 phosphorylation. However, pharmacological inhibition of ERK1/2 activity may abolish the protective effect offered by RIPC. This RIPC-mediated testicular protection is executed by the activation of the ERK1/2-dependent signaling pathway.

## Supporting information

S1 File (PDF)
