# Supplementary material for: Pretreatment with remote ischemic conditioning attenuates testicular damage after testicular ischemia and reperfusion injury in rats
Source: PLoS One. 2023 Oct 26;18(10):e0287987. doi: 10.1371/journal.pone.0287987 (PMC10602300; doi:10.1371/journal.pone.0287987)
Supplement: S1 File — (PDF) [file pone.0287987.s001.pdf]

We tested the testicular expression levels of phosphorylated ERK1/2 and total ERK1/2 in our study. Both proteins have same molecular weight and were probed on the same location in the same membrane. We first detected the expression level of phosphorylated ERK1/2, and then stripped the membrane. We then probed the same membrane with total ERK1/2. The phosphorylation levels of ERK1/2 were normalized with total ERK1/2.

Figure 8A

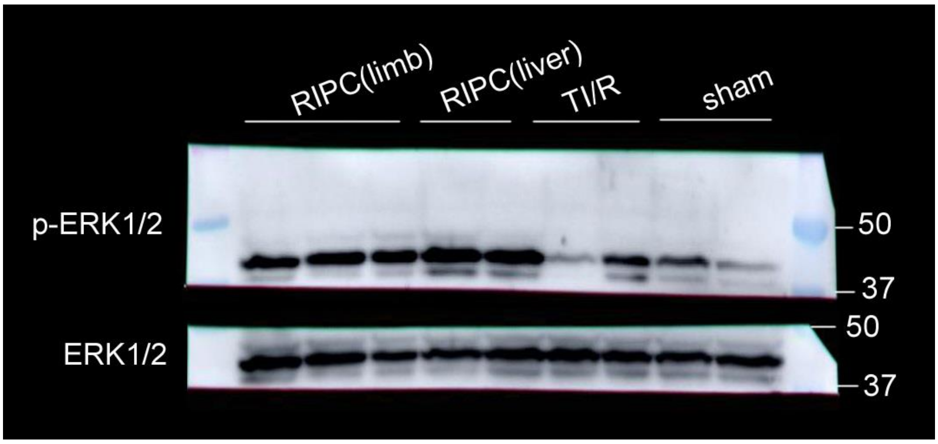

Figure 8B

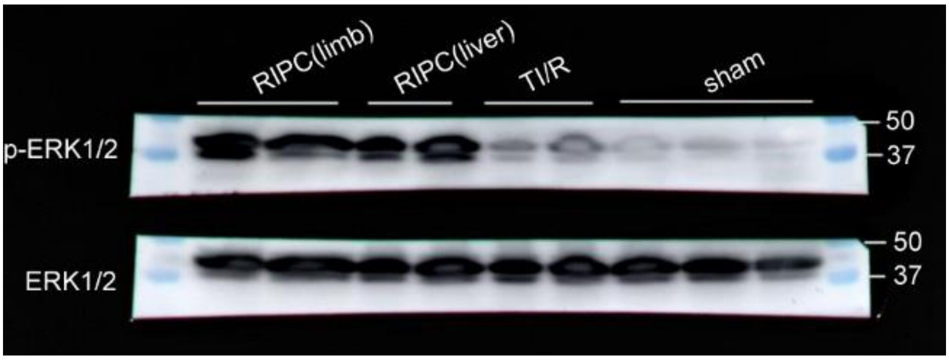

Figure 10A

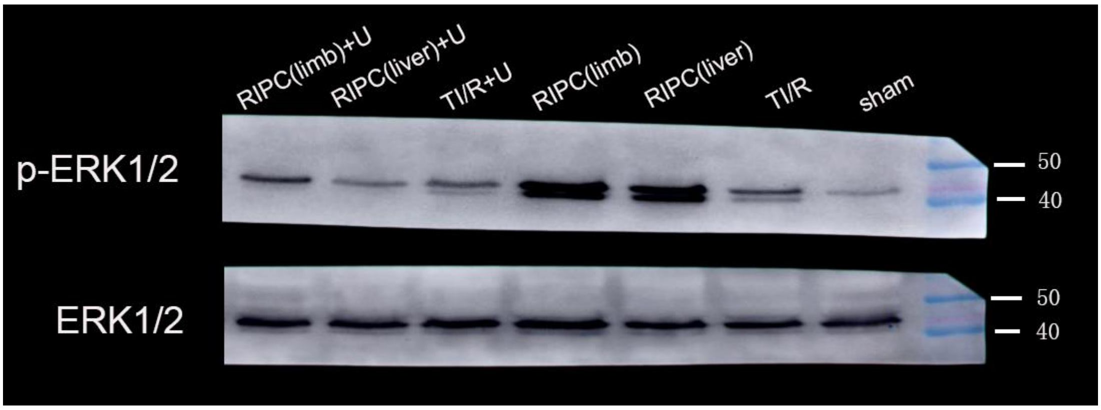
RIPC(limb)+U
RIPC(liver)+U
TI/R+U
RIPC(limb)
RIPC(liver)
TI/R
sham
p-ERK1/2
ERK1/2
50
40
50
40

Figure 10B

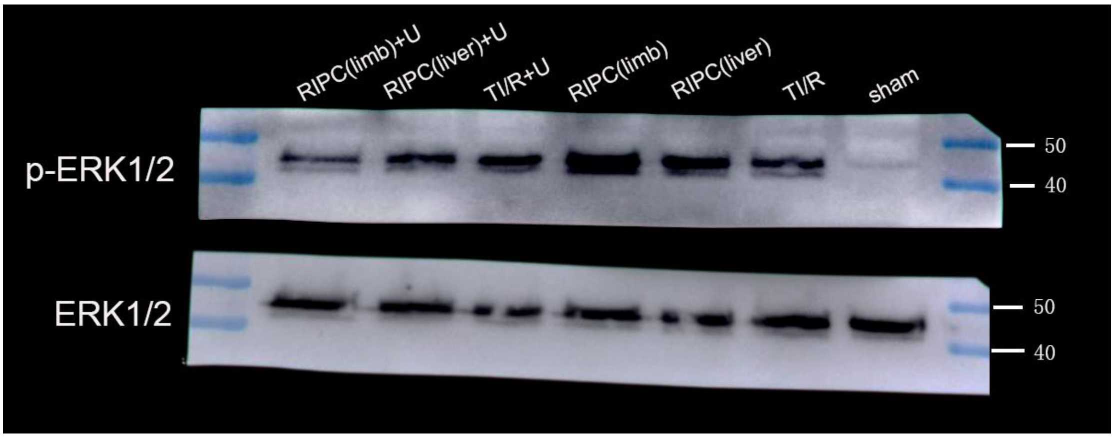
RIPC(limb)+U
RIPC(liver)+U
TI/R+U
RIPC(limb)
RIPC(liver)
TI/R
sham
p-ERK1/2
ERK1/2
50
40
50
40
